# Supplementary material for: Mode coupling bi-stability and spectral broadening in buckled carbon nanotube mechanical resonators
Source: Nat Commun. 2022 Oct 6;13:5900. doi: 10.1038/s41467-022-33440-4 (PMC9537592; doi:10.1038/s41467-022-33440-4)
Supplement: Supplementary file 1 — Supplementary Information [file 41467_2022_33440_MOESM1_ESM.pdf]

Mode coupling bi-stability and spectral broadening in buckled nanotube mechanical resonators

Supplementary information

S. Rechnitz[*†], T. Tabachnik[*†], M. Shlafman[†], S. Shlafman[†], and Y. E. Yaish[†]

[†]Andrew and Erna Viterbi Faculty of Electrical Engineering, Technion, Haifa 32000, Israel.

*These authors contributed equally to this work.

Supplementary text contents:

1. Fabrication and control of buckled CNT resonators
2. Influence of initial buckling
3. Snap-through buckling evidence in conductance measurements
4. Theoretical modelling
5. Linearization justification
6. Calculation of the resonance frequencies and spectral broadening
7. Fluctural broadening calculation at varying temperatures
8. Nonlinear oscillator equation and symmetry breaking
9. Physical parameters table

1. Fabrication and control of buckled CNT resonators

The fabrication process is detailed in the Methods section. The BOE etching is isotropic. Therefore, in addition to creating the trench between the source and drain electrodes, it also creates a small undercut underneath them. Then, another step of evaporation is performed, such that the final SD electrodes consist of a Cr/Pt/Cr/Pt 5nm/35nm/5nm/35nm heterostructure, with suspended edges. At the final stage of the CVD growth, the sample is heated to a temperature of 900°C, and thus the suspended metallic parts of the heterostructure will deform at the edges, due to thermal expansion coefficient mismatch. We believe that this deformation causes the CNT to favor upward buckling during the growth. To support our hypothesis, we examined the BOE etch depth vs the device classification (Fig. S1) and the device classification vs SD deformation (Fig. S2). Although the yield is not 100%, both graphs together with Fig. S3 demonstrate that we can control the initial buckling relatively well by the BOE etch step of the fabrication.

Note: The data in Figs. S1 and S2 is based on 12 devices in the 1st category, 8 devices in the 2nd category, 17 devices in the 3rd category and 14 devices in the 4th category.

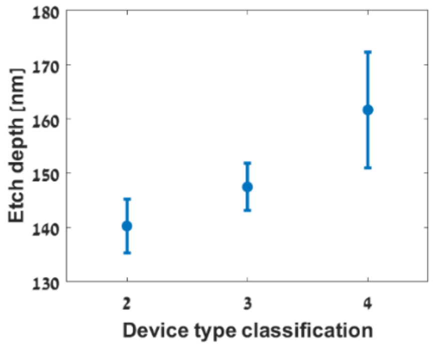

Fig. S1. BOE effect on the device classification. The effect of the BOE etching depth (depth measured via AFM, reflecting on the etch time) on the classification of the device, for upward buckled CNT resonators. Data taken from all fabricated devices, most of which are not presented in the article.

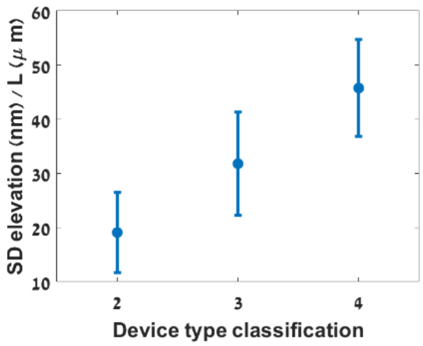

Fig. S2. Contacts deformation effect on the device classification. The effect of Source and Drain deformation (measured in AFM, normalized by the device length) on the classification of the device for upward buckled CNT resonators.

2. <u>Influence of initial buckling</u>

As explained in the main text (Fig. 2), the initial configuration of the CNT determines which type of behavior is to be expected. To support this claim, Fig. S3 presents the fitted b_0 vs classification for the devices in Fig. 2. One can clearly observe that as b_0 decreases (absolute value increases), the classification changes according to our qualitative explanation in the main text (Fig. 2).

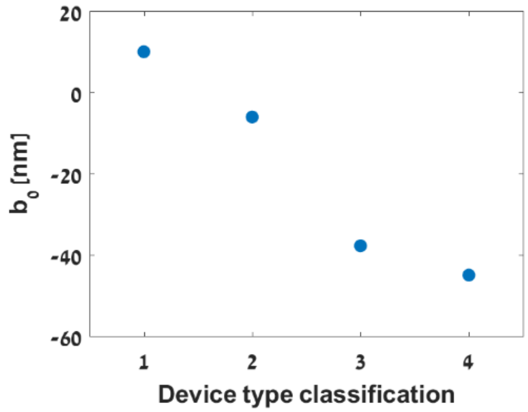

Fig. S3. Correlation between initial midpoint elevation and the device classification. Initial midpoint elevation extracted from the fitting for the devices presented in Fig. 2, divided by their classification.

We shall note that for the fitting, we use the physical parameters of each device (CNT diameter, length, trench depth) as extracted from an AFM image of the device. However, the initial configuration of the CNT is a fitting parameter. Only after fitting we can extract the theoretical b_0.

Therefore, to establish that the extracted initial buckling from the fitted data agrees with the real initial CNT displacement, we present a comparison of the theoretical b_0 with the SEM images of two devices in the third category (Fig. S4). From the SEM image in Fig. S4a we extracted initial midpoint elevation of b_0=47±2nm, and from the theoretical fitting (Fig. S4c) we extracted initial midpoint elevation of b_0=42nm. From the SEM image in Fig. S4d we extracted initial midpoint elevation of b_0=43±4nm, and from the theoretical fitting (Fig. S4f) we extracted initial midpoint elevation of b_0=37.8nm. We shall point out that the typical buckling heights are less than 5% of the tube length and are hence hard to notice in the SEM images.

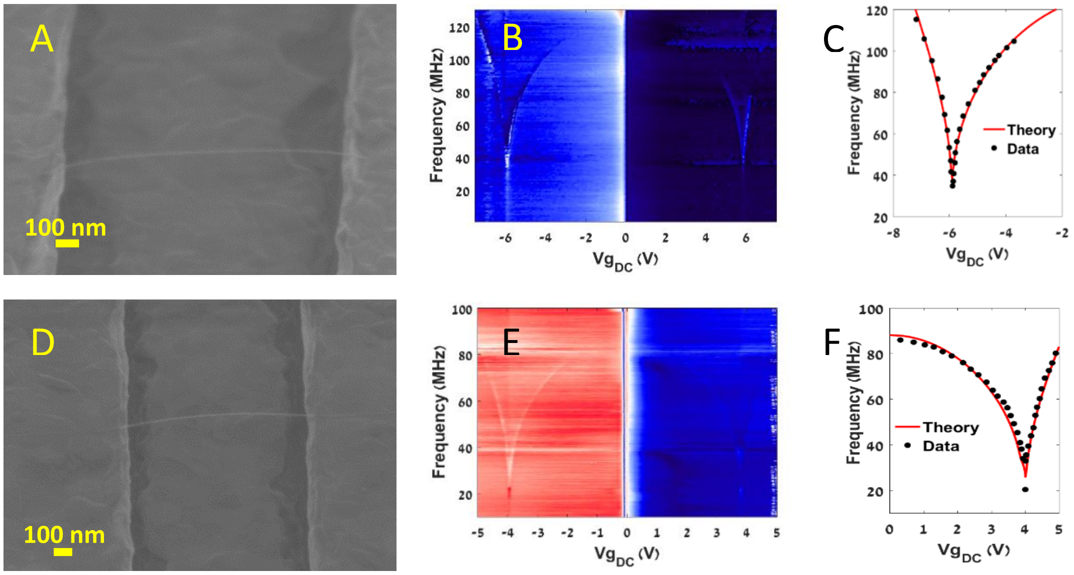

Fig. S4. Experimental verification of the theoretical prediction for b_0. (a,d) SEM images of initially buckled upward CNTs (devices X and V, respectively), retrieved at a 75º and 70º (respectively) angle from the perpendicular to the surface. (b,e) Resonance frequency measurements of the same devices as in (a) and (d), respectively, typical of the third category (obtained prior to the SEM measurements). Measurement (e) is the same as Fig. 2c. (c,f) Theoretical fitting (red line) to the data (black dots) extracted from the resonance measurement in (b,e), respectively.

3. Snap-Through Buckling Evidence in Conductance Measurements

The physical configuration of the CNT directly affects the capacitance between the CNT and the local-gate. Therefore, the snap-through (ST) and release transitions should be accompanied by capacitance modification. This change in the capacitance will modulate the charge on the CNT, translating into a change in the CNT conductance. Hence, at the unstable ST point, the abrupt mechanical transition causes a noticeable discontinuity in the conductance measurements, characterized also by hysteresis. Fig. S5 presents the transfer characteristics curve of device II. Along with the common small band gap behavior, we observe additional features of discontinuities and hysteresis (marked by the dashed lines), corresponding to the resonance "jumps" in Fig. 1d.

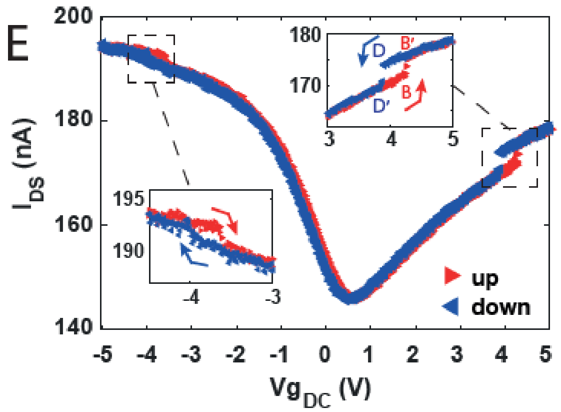

Fig. S5. ST buckling evidence in DC conductance measurement. Conductance measurement of device II, exhibiting the snap-through and release phenomena. Insets: Zoom-in on areas marked by dashed rectangles.

4. Theoretical Modelling

To analyze the static response of the system, we begin from the following relations of the force moment, $\mathbf{M}$[1]:

$$\mathbf{M} = EI\mathbf{t} \times \frac{d\mathbf{t}}{dl} + \mathbf{t}GJ\tau$$

$$\frac{d\mathbf{M}}{dl} = \mathbf{F} \times \mathbf{t} \qquad \text{S1}$$

where E is the CNT Young's modulus, I is the moment of inertia, $\mathbf{t}$ is the unit vector tangential to the CNT ($\mathbf{t} = \frac{d\mathbf{r}}{dl}$), G is the shear modulus, J is the polar moment of inertia, and $\tau = \Omega \cdot \mathbf{t}$ where Ω is the moment of the forces acting on a cross section of the tube, thus $\tau = \frac{d\varphi}{dl}$ is the twisting component in the tangential direction and $\varphi(l)$ is the twist angle along the tube.

Taking the second derivative $d^2\mathbf{M} / dl^2$ results in:

$$EI\frac{d\mathbf{t}}{dl} \times \frac{d^2\mathbf{t}}{dl^2} + EI\mathbf{t} \times \frac{d^3\mathbf{t}}{dl^3} + GJ\tau\frac{d^2\mathbf{t}}{dl^2} + 2GJ\frac{d\tau}{dl}\frac{d\mathbf{t}}{dl} + GJ\frac{d^2\tau}{dl^2}\mathbf{t} = \frac{d\mathbf{F}}{dl} \times \mathbf{t} + \mathbf{F} \times \frac{d\mathbf{t}}{dl} \qquad \text{S2}$$

Substituting $\frac{d\mathbf{F}}{dl} = -\boldsymbol{\kappa}$, where $\boldsymbol{\kappa} = \kappa_z$ the external force (acting in the z direction only), assuming $dl \simeq dx$ (shallow arch) and separating into x,y,z components yields the following set of equations which describe the CNT static motion:

$$\hat{x} : EI\left(y'z'''' - z'y'''' + y''z''' - z''y'''\right) + GJ\varphi''' = y'\kappa_z - F_z y''$$

$$\hat{y} : EIz'''' - GJ\left(\varphi'y''' + 2\varphi''y'' + \varphi'''y'\right) = \kappa_z - Pz'' + \frac{EA}{2L}z''\int_0^L\left(z'^2 + y'^2 + r^2\varphi'^2\right)dx \qquad \text{S3}$$

$$\hat{z} : EIy'''' + GJ\left(\varphi'z''' + 2\varphi''z'' + \varphi'''z'\right) = -Py'' + \frac{EA}{2L}y''\int_0^L\left(z'^2 + y'^2 + r^2\varphi'^2\right)dx$$

where we substituted $F_x = -P + \frac{EA}{2L}\int_0^L\left(z'^2 + y'^2 + r^2\tau^2\right)dx$, the tension along the tube, in which P is the initial axial tension at $V_g^{DC} = 0$. $z(x,t), y(x,t)$ are the in-plane and out-of-plane deflections along the beam, respectively, and $\varphi(x,t)$ is the twist angle along the tube. A is the CNT cross-

section area, L is the horizontal distance between the clamped ends of the suspended CNT, r is the CNT radius and κ_z is the electrostatic force exerted by the local-gate, calculated according to: $\kappa_z = \frac{1}{2}\frac{\partial C_g}{\partial z}\left(V_{gDC} + V_{gAC}\right)^2$. We assume $V_{gAC} \ll V_{gDC}$ so $\left(V_{gDC} + V_{gAC}\right)^2 \approx V_{gDC}^2 + V_{gDC}V_{gAC}$ and use the first term as the static force and the second term for the harmonic actuation. The capacitance is calculated according to a "wire parallel to plane" approximation, which, under the assumption $\frac{(g_0+w)^2}{r^2} \gg 1$, can be approximated as $C_g(z) = \frac{2\pi\varepsilon_0}{\ln\left(\frac{2(g_0+z)}{r}\right)} \Rightarrow \frac{\partial C_g}{\partial z}(z) = \frac{\pi\varepsilon_0}{(g_0+z)\left(\ln\left(\frac{2(g_0+z)}{r}\right)\right)^2}$.

For the dynamic response analysis, we add the acceleration term to the $\hat{y}, \hat{z}$ equations to receive the Euler-Bernoulli beam equations. For the torsional vibrations we use the first derivative $\frac{d\mathbf{M}}{dl} - \mathbf{F} \times \mathbf{t} = \rho I_x \frac{\partial^2 \varphi}{\partial t^2}$. Finally, we can describe the dynamic motion of the CNT by the following set of equations:

$$\begin{aligned} &\hat{x}: EIy'z''' - EIz'y''' + GJ\varphi'' + F_z y' - \rho I_x \ddot{\varphi} = 0 \\ &\hat{y}: EIz'''' - GJ\left(\varphi' y''' + 2\varphi'' y'' + \varphi''' y'\right) + \rho A\ddot{z} - \kappa_z + Tz'' = 0 \\ &\hat{z}: EIy'''' + GJ\left(\varphi' z''' + 2\varphi'' z'' + \varphi''' z'\right) + \rho A\ddot{y} + Ty'' = 0 \end{aligned} \qquad \text{S4}$$

where t is time, I_x is the torsional moment of inertia, ρ is the mass density, and T is the tension, given by $T = -P + \frac{EA}{2L}\int_0^L \left(z'^2 + y'^2 + r^2\varphi'^2\right)dx$, as before.

We define the tube's deflection as a superposition of the initial buckling (subscript 0), the static deflection due to the DC voltage (subscript s) and the dynamic oscillation due to the AC actuation (subscript d), as illustrated for the in-plane component in Fig. S6:

$$\begin{aligned} z(x,t) &= z_0(x) + z_s(x) + z_d(x,t) \\ y(x,t) &= y_0(x) + y_s(x) + y_d(x,t) \\ \varphi(x,t) &= \varphi_0(x) + \varphi_s(x) + \varphi_d(x,t) \end{aligned}$$

Doubly clamped boundary conditions were imposed.

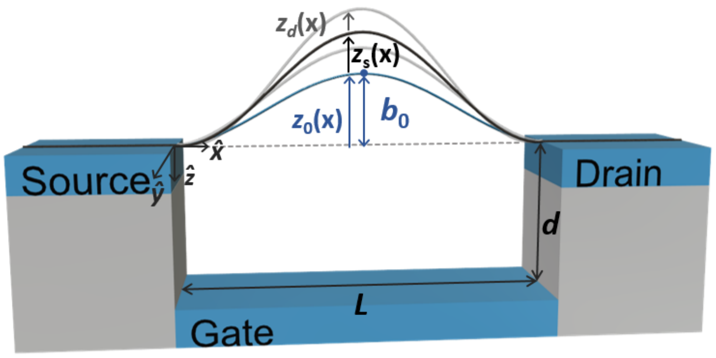

Fig. S6. Device geometry. Schematic illustration for a doubly clamped suspended CNT with in-plane movement, represented by the superposition of the initial buckling (z_0), static deflection (z_s) and dynamic deflection (z_d).

Eqs. S3 and S4 are nonlinear integro-differential equations, and therefore finding an exact analytical solution is difficult. However, by discretizing the system into finite degrees of freedom using a reduced order model (ROM) instead of solving a continuous system, an approximated solution can be found. For this task, we chose the Galerkin method[2], in which the beam deflection is approximated as a linear combination of eigenmodes of the linear Euler-Bernoulli equation for a doubly clamped straight beam, noted by $\xi_i(x)$:

$$z(x,t)=\sum_{i=1}^{n} q_i(t)\xi_i(x)=\sum_{i=1}^{n}\left[q_{0,i}+q_{s,i}+q_{d,i}(t)\right]\xi_i(x)$$
$$y(x,t)=\sum_{i=1}^{m} v_i(t)\xi_i(x)=\sum_{i=1}^{n}\left[v_{0,i}+v_{s,i}+v_{d,i}(t)\right]\xi_i(x)$$
$$\varphi(x,t)=\sum_{i=1}^{m} \varphi_i(t)\xi_i(x)=\sum_{i=1}^{n}\left[\varphi_{0,i}+\varphi_{s,i}+\varphi_{d,i}(t)\right]\xi_i(x)$$

The discretization is therefore realized by taking a finite number of eigenmodes. Specifically, by comparing with a finite element method solution, we find that only the first two modes suffice:

$$z_0(x)=q_{01}\xi_1(x)+q_{02}\xi_2(x) \qquad y_0(x)=v_{01}\xi_1(x)+v_{02}\xi_2(x) \qquad \varphi_0(x)=\varphi_0 x+\varphi_{01}\xi_1(x)+\varphi_{02}\xi_2(x)$$
$$z_s(x)=q_{1s}\xi_1(x)+q_{2s}\xi_2(x) \qquad y_s(x)=v_{1s}\xi_1(x)+v_{2s}\xi_2(x) \qquad \varphi_s(x)=\varphi_{1s}\xi_1(x)+\varphi_{2s}\xi_2(x)$$
$$z_d(x,t)=q_{1d}(t)\xi_1(x)+q_{2d}(t)\xi_2(x) \qquad y_d(x,t)=v_{1d}(t)\xi_1(x)+v_{2d}(t)\xi_2(x) \qquad \varphi_d(x,t)=\varphi_{1d}(t)\xi_1(x)+\varphi_{2d}(t)\xi_2(x)$$

This process transforms Eqs. S3 and S4 into a set of algebraic equations, which are solved numerically. We solve Eqs. S3 for the initial buckling and static motion ($q_0, q_s, v_0, v_s, \varphi_0, \varphi_s$) and

only then solve Eqs. S4 for the resonance frequencies (i.e., the eigenvalues of the homogeneous system of equations) at the specific static position.

We shall emphasize that the CNT initial configuration is not restricted to be solely in-plane. We allow initial out-of-plane deflection by taking $y_0 \neq 0$. Nonetheless, our SEM images and theoretical fitting reveal that the out-of-plane initial buckling is much smaller compared to the in-plane buckling ($y_0 << z_0$). Hence, the qualitative differentiation between device types based solely on the in-plane b_0 in Fig. 2 is sufficient. Quantitatively, in order to achieve the good fitting, we must include non-zero out-of-plane deflection, even if it is small.

Fig. S7 presents a SEM image of the same device as Fig. S4a, taken perpendicular to the surface (from above), in which the CNT appears nearly straight, meaning that the out-of-plane component of the initial beam shape (y_0) is very small compared to the in-plane component (z_0) apparent in Fig. S4a.

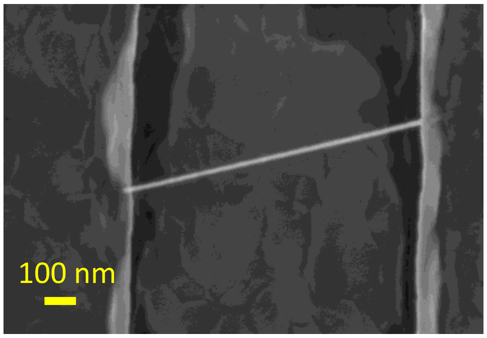

Fig. S7. SEM image of a typical buckled device. Top view SEM image of the same CNT as in Fig. S4a, retrieved perpendicular to the sample surface. The beam appears straight, implying that the out-of-plane component of the initial beam shape (y_0) is much smaller than the in-plane curvature (z_0) apparent in Fig. S4a.

5. <u>Linearization justification</u>

Let us first explain the origin of the non-linearity in the Euler-Bernoulli (EB) beam equation, and to clarify which terms are still linear.

Let's assume that we have a CNT along the x direction and its movements can be either in-plane (x-z plane) or out-of-plane (x-y plane). See Fig. 1a, which depicts the geometry and notation of our problem.

For our purpose, it is sufficient to limit ourselves to the static deflection (the conclusions for the dynamic case are the same). Let's assume in the beginning that the motion is restricted to the x-z plane and note the static deflection along the z direction by *w*(x). Assuming initial compression (-T_0<0) and induced tension which arises from the shape of the beam, $T_{ind}(w(x))$, we end up with the following EB equation:

$$EI\frac{d^4}{dx^4}w(x)+T_0\frac{d^2}{dx^2}w(x)-T_{ind}\frac{d^2}{dx^2}w(x)=F_{ext} \qquad \text{S5}$$

where E is the tube Young's modulus, I is the moment of inertia, and F_{ext} is the external static force per unit length. The non-linearity of this equation arises from two terms. The first is expressed by T_{ind} and the second is related to the external force.

Let's examine first T_{ind}. When the tube is not straight the deflection *w(x)* makes the overall length (L) longer than its relaxed length, L_0. Therefore, the induced tension will be given by: $T_{ind} = EA\frac{\Delta L}{L_0} = EA\frac{(L-L_0)}{L_0}$, where A is the tube's cross section. How shall we calculate L? We look at two adjacent deflections along the tube: *w(x)* and *w(x+dx)*. The length of the tube's segment from x to $x+dx$, is given by $dl = \sqrt{dx^2+dw^2} = dx\sqrt{1+\left(\frac{dw}{dx}\right)^2}$. Since the tube length is much bigger than its maximal deflection (w_{max}/L_0 ~ 50nm/1000nm = 1/20), we can expand the square root and approximate dl: $dl = dx\sqrt{1+\left(\frac{dw}{dx}\right)^2} \approx dx\left(1+\frac{1}{2}\left(\frac{dw}{dx}\right)^2\right)$. If, for example, we assume that *w(x)* is the first buckling solution of the EB equation $w(x) = \frac{1}{40}\left(1-\cos(2\pi x/L_0)\right)$ which satisfies the requirement that max($w(x)$)/L_0=$w(x=L_0/2)/L_0$=1/20, we can calculate the error in this approximation –

The total length of the deformed tube is given by:

$$L=\int_0^{L_0}dx\sqrt{1+\left(\frac{dw}{dx}\right)^2}=\int_0^{L_0}dx\sqrt{1+\left(\frac{2\pi}{40}\sin(2\pi x/L_0)\right)^2} \qquad \text{S6}$$

The approximate expression (L_{app}):

$$L_{app}=\int_0^{L_0}dx\left(1+\frac{1}{2}\left(\frac{dw}{dx}\right)^2\right)=\int_0^{L_0}dx\left(1+\frac{1}{2}\left(\frac{2\pi}{40}\sin(2\pi x/L_0)\right)^2\right) \qquad \text{S7}$$

The relative error between the two lengths is $\frac{|L-L_{app}|}{L}=3\cdot 10^{-5}$, which verify the validity of this approximation for our devices. If we return to the definition of the induced tension, we find:

$$T_{ind}=EA\frac{L-L_0}{L_0}=EA\left(\frac{1}{L_0}\int_0^{L_0}dx\sqrt{1+\left(\frac{dw}{dx}\right)^2}-1\right)\approx\frac{EA}{2L_0}\int_0^{L_0}dx\left(\frac{dw}{dx}\right)^2 \qquad \text{S8}$$

After inserting this expression into Eq. S6 we find the source of the geometric non-linearity. The term $T_{ind}w''$ is an integro-differential non-linear term but the differential terms which are not within the integral are all linear. Hence, the linearity of the differential equation is preserved, and using the superposition principle is valid.

Now, let us examine what happens if the motion is not restricted to the x-z plane. Figure S8 presents two segments of a circle. The z axis marked as *w* (in-plane deflection), and the y axis marked as *v* (out of plane deflection). The longitudinal direction remains *x*. The red segment presents the situation in which the deflection is solely within the x-z (or x-w) plane (red arrow). Then, we rotate the red segment by 45° and end up with the blue segment. At this specific configuration, if we wish to describe the segment (or tube) deformation, we can use the red deformation and rotate it by θ=45°. Now, the description of the tube deformation consists of *w(x)* (x-axis) as well as *v(x)* (y-axis). Mathematically, we can express the relation between the red and blue deformations as the following:

$$\begin{aligned} w(x)&=w(x)\cdot\cos(\theta)\\ v(x)&=-w(x)\cdot\sin(\theta)\end{aligned} \qquad \text{S9}$$

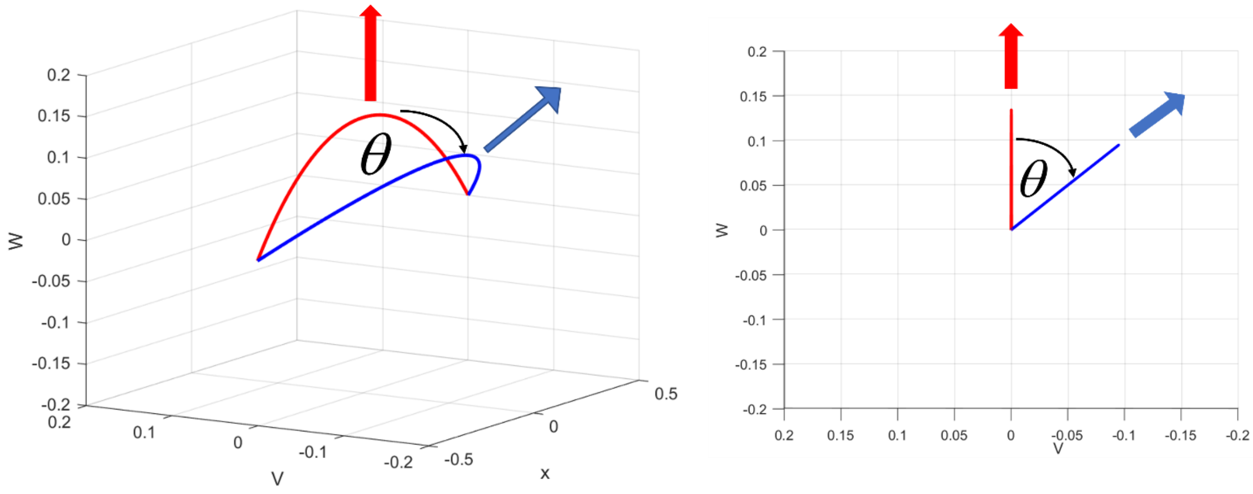

Fig. S8. **The definition of rotation angle.** Two circular segments with a relative θ=45° rotation with respect to each other. left – 3D plot, right – projection on to the *w*-*v* (z-y) plane at *x*=0 (segment center).

Let us calculate the induced tension of the blue segment, and let's assume that the angle of rotation is general (θ):

$$T_{ind} = \frac{EA}{2L_0}\int_0^{L_0} dx\left\{\left(\frac{dw}{dx}\right)^2 + \left(\frac{dv}{dx}\right)^2\right\} = \frac{EA}{2L_0}\int_0^{L_0} dx\left\{\left(\frac{dw}{dx}\cos(\theta)\right)^2 + \left(\frac{dw}{dx}\sin(\theta)\right)^2\right\} = \frac{EA}{2L_0}\int_0^{L_0} dx\left(\frac{dw}{dx}\right)^2\left(\cos^2(\theta)+\sin^2(\theta)\right) = \frac{EA}{2L_0}\int_0^{L_0} dx\left(\frac{dw}{dx}\right)^2 = T_{ind} \quad \text{S10}$$

As expected, since the circular shape remains the same, the induced tension should be identical. One can think about it as rotation of the reference coordinate system by -θ (minus θ). In other words, we could describe the circular deflection with vectorial deflection:

$$\vec{\psi}(x) = w(x)\hat{z} + v(x)\hat{y} \quad \text{S11}$$

and the EB equation can be written as follows:

$$EI\frac{d^4}{dx^4}\vec{\psi}(x) + T_0\frac{d^2}{dx^2}\vec{\psi}(x) - T_{ind}\frac{d^2}{dx^2}\vec{\psi}(x) = \vec{F}_{ext} \quad \text{S12}$$

where $\boldsymbol{F}_{ext}$ is the external force in the z and y direction (in our case the external force is solely in the z direction), and the induced tension is given by:

$$T_{ind} = \frac{EA}{2L_0}\int_0^{L_0} dx\frac{d\vec{\psi}}{dx}\cdot\frac{d\vec{\psi}}{dx} = \frac{EA}{2L_0}\int_0^{L_0} dx\left\{\left(\frac{dw}{dx}\right)^2 + \left(\frac{dv}{dx}\right)^2\right\} \quad \text{S13}$$

If we decompose Eq. S12 to its components along the z and y directions, we find:

$$\hat{z}: \quad EI\frac{d^4}{dx^4}w(x)+T_0\frac{d^2}{dx^2}w(x)-T_{ind}\frac{d^2}{dx^2}w(x)=F_{ext}^z \qquad \text{S14}$$

$$\hat{y}: \quad EI\frac{d^4}{dx^4}v(x)+T_0\frac{d^2}{dx^2}v(x)-T_{ind}\frac{d^2}{dx^2}v(x)=F_{ext}^y \qquad \text{S15}$$

To conclude, the induced tension is an important term. It is responsible for both the non-linearity of the EB equation, and for the coupling between the in-plane and out-of-plane motions.

This non-linear term is the origin for the Duffing oscillator in nano-beams, and responsible for numerous non-linear effects, such as parametric amplification, modes-coupling, and of-course the EB buckling instability and the snap-through transition. In our analysis we imply the super position principle only for the linear terms, where for the non-linear terms we use the full expression without omitting any term.

The second source of non-linearity arises from the external electric force of the local gate, which also depends on the tube deflection along the z-direction. This non-linearity was taken as well, and no super position principle was implied to this term.

6. Calculation of the resonance frequencies and spectral broadening

As explained in the main text, the built-in strain along the nanotube affects its resonance frequencies. Since the resonance frequencies of the out-of-plane modes are always lower than the in-plane modes, we expect that the thermal fluctuations of the out-of-plane modes $\sigma_{x_{op}^2}=\frac{k_B T}{m\omega_{op}^2}$ will affect both the in-plane resonance frequencies, f_{ip}, and their spectral broadening, Δf_{ip} [3]. Throughout the mode coupling discussion, we shall neglect the coupling to the torsional modes since, as explained in the main text, their resonance frequencies are much higher and therefore their effect on the in-plane modes is negligible.

It is customary to transform equations S3 and S4 to be dimensionless. We normalize the equations according to $x\to\frac{x}{L}$, $y\to\frac{y}{d}$, $z\to\frac{z}{d}$, $r\to\frac{r}{d}$, $t\to\frac{t}{t_0}$, where d is the trench depth and $t_0\triangleq\sqrt{\frac{\rho AL^4}{EI}}$, so the out-of-plane motion is represented by the dimensionless parameter $v_d=\frac{y}{d}$.

The dimensionless algebraic in-plain equations include coupling between the in-plane and out-of-

plane modes in the form of $\propto q_d \left(v_{1d}^2 \int \xi_1'^2 + v_{2d}^2 \int \xi_2'^2 + ... \right)$, expressing the built-in strain resulting from the vibrations of the out-of-plane modes.

Barnard *et al.* show in Ref. 3 that for a buckled beam, the in-plane resonance fluctuations, σ_f, are related to the thermal fluctuations of the out-of-plane mode, $\sigma_{y^2} = \frac{k_B T}{m\omega_{op}^2}$, according to:

$$\sigma_f = \frac{1}{2\pi} \left| \frac{\partial \omega_{ip}}{\partial y^2} \right| \cdot \sigma_{y^2} \qquad \text{S16}$$

where k_B is the Boltzmann constant, T is temperature and $m\omega_{op}^2$ is the effective spring constant of the out-of-plane mode.

Our theoretical model allows us to calculate both the out-of-plane resonance frequencies, and the derivative $\left| \frac{\partial \omega_{ip}}{\partial v_d^2} \right|$ for every static load, according to $\left| \frac{\partial \omega_{ip}}{\partial v_d^2} \right| = \frac{1}{2\omega_{ip}} \left(\frac{\partial \omega_{ip}^2}{\partial v_d^2} \right)$.

Substituting these results into Eq. S16 and using the relation $\Delta f_{FWHM} \sim 0.65\sigma_f$ yields the excellent fit for device IX in Fig. 4. Another example of a good fit for a second CNT (device I, Fig. 1c) is presented in Fig. S11.

One should wonder how dominant is the lowest out-of-plane mode compared to higher out-of-plane modes as well as higher in-plane modes[4]. To take into account an additional out-of-plane mode, we simply sum their contributions according to:

$$\sigma_{y^2} = \sqrt{\left(\frac{k_B T}{m\omega_{op1}^2} \right)^2 + \left(\frac{k_B T}{m\omega_{op2}^2} \right)^2} \qquad \text{S17}$$

It turns out that the contribution of the second mode is negligible (Fig. S9). This result is not surprising since the lowest out-of-plane resonance frequency is very close to the lowest in-plane mode, whereas the second out-of-plane and second in-plane resonance frequencies are significantly higher. For example, at V_g=0, $\omega^2_{op2}/\omega^2_{op1}$=17.52 and $\omega^2_{ip2}/\omega^2_{op1}$=18.12, and at the inflection point (V_g=5.532V) $\omega^2_{op2}/\omega^2_{op1}$=25.55 and $\omega^2_{ip2}/\omega^2_{op1}$=26.76.

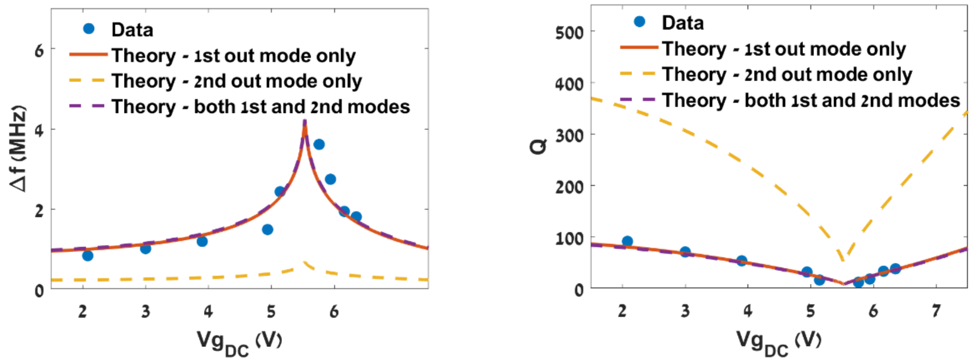

Fig. S9. Spectral broadening analysis at room temperature. Spectral broadening (FWHM, left) and quality factor (right) extracted from Fig. 4a vs the static gate load (blue dots). The orange solid line is the theoretical calculation based on Eq. 1, considering only the 1st out-of-plane mode. The dashed yellow line is the theoretical calculation, considering only the 2nd out-of-plane mode. And the dashed purple line is the theoretical calculation summing the contribution of both the 1st and 2nd out-of-plane modes. The excellent fit implies that fluctuation broadening is the most significant dissipation mechanism in the system, and the similarity between the orange and purple lines implies that the coupling to the first mode governs the broadening.

7. Fluctural broadening calculation at varying temperatures

Since fluctuation broadening is based on the equipartition theorem, the broadening depends on temperature. Hence, to further investigate this theory, we decided to repeat the same measurements and spectral broadening analysis at varying temperatures. At each temperature, we performed a resonance frequency measurement vs gate voltage (example obtained at $T=38^{o}$K is presented in Fig. S10a). From the theoretical fitting to this data (Fig. S10b), we calculated the strain gate voltage dependence at the different temperatures. Then we used the same methodology for calculating the theoretical broadening due to the first out-of-plane mode using Eq. 1. The experimental spectral broadening was extracted from a general Lorentzian fit to the resonance peak measured at each temperature (Fig. S10a inset). The comparison between the broadening extracted directly from the measurement and the broadening estimated according to the theory at the same gate voltage and temperature is presented in Fig. S10c. The compatibility between experiment and theory is quite remarkable.

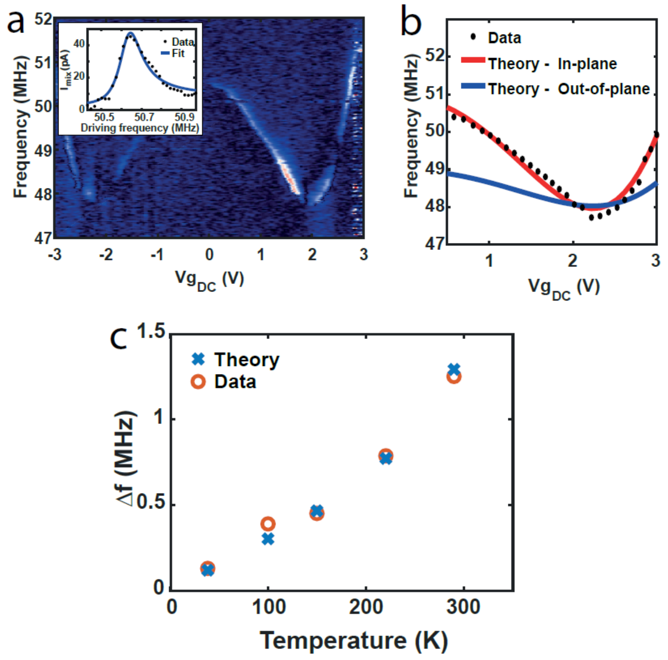

Fig. S10. Temperature-dependent spectral broadening analysis. (a) Resonance frequency measurement of device IV in the second category, obtained at T=38°K. Inset is a frequency cross section obtained at V_{gDC}=0.8V, from which the left-most data mark in (c) was extracted. Black dots are the data, blue solid line is a Lorentzian shape fit. (b) Modelling the dynamic response of the measurement in (a), from which the left-most theoretical mark in (c) was calculated. The solid lines are the theoretical fitting to the experimental data (black dots). (c) Comparison between the experimental spectral broadening (FWHM) extracted from resonance peaks measured at varying temperatures from device IV (orange marks) vs. the theoretical prediction according to Eq. 1 (blue marks).

8. Nonlinear oscillator equation and symmetry breaking

For this discussion, we shall take only the first in-plane mode and neglect the out-of-plane and torsion coupling terms. In this case, if we add the damping term, $\gamma\frac{\partial z_d}{\partial t}$, the dynamic beam equation for the in-plane modes is:

$$EI\frac{\partial^4 z_d}{\partial x^4}+\rho A\frac{\partial^2 z_d}{\partial t^2}+\gamma\frac{\partial z_d}{\partial t}-\left(-P+\frac{EA}{2L}\int_0^L\left(\frac{\partial z_0}{\partial x}+\frac{\partial z_s}{\partial x}\right)^2 dx\right)\frac{\partial^2 z_d}{\partial x^2}-\left(\frac{EA}{2L}\int_0^L\left[2z_0'z_d'+2z_s'z_d'+z_d'^2\right]dx\right)\frac{\partial^2 z}{\partial x^2}=\kappa_d$$

where κ_d is the dynamic force exerted by the local-gate.

Substituting $z_0(x)=q_{01}\xi_1(x)$, $z_s(x)=q_{1s}\xi_1(x)$, $z_d(x,t)=q_{1d}(t)\xi_1(x)$, multiplying the equation by $\xi_1(x)\cdot L$ and integrating over the beam length results in:

$$\frac{EI}{L^2}q_{1d}\int\xi_1''\xi_1''+\rho AL\frac{d^2q_{1d}}{dt^2}\int\xi_1^2+cL\frac{dq_{1d}}{dt}\int\xi_1^2-\left(-P+\frac{EA}{2L^2}(q_{01}+q_{1s})^2\int\xi_1'\xi_1'\right)q_{1d}\int\xi_1'\xi_1'$$
$$+\frac{EA}{2L^2}\left(\int\xi_1'\xi_1'\right)^2(q_{01}+q_{1s}+q_{1d})\left(2(q_{01}+q_{1s})q_{1d}+q_{1d}^2\right)=\kappa_{zd}\int\xi_1 \qquad \text{S18}$$

Rearranging the terms in Eq. S8 to the powers of $q_{1d}(t)$ and its derivatives will lead to the following equation:

$$\rho AL\int \xi_1^2 \cdot \frac{d^2q_{1d}}{dt^2} + cL\int \xi_1^2 \cdot \frac{dq_{1d}}{dt} + \left[\frac{EI}{L^2}\int \xi_1^{''}\xi_1^{''} - P\int \xi_1^{'}\xi_1^{'} + \frac{3EA}{2L^2}\left(\int \xi_1^{'}\xi_1^{'}\right)^2 (q_{01}+q_{1s})^2\right] q_{1d}$$
$$+\frac{3EA}{2L^2}\left(\int \xi_1^{'}\xi_1^{'}\right)^2 (q_{01}+q_{1s}) q_{1d}^2 + \frac{EA}{2L^2}\left(\int \xi_1^{'}\xi_1^{'}\right)^2 q_{1d}^3 = \kappa_{zd}\int \xi_1 \quad \text{S19}$$

By defining $u(t) \equiv q_{1d}(t)$ and the following coefficients:

$$m = \rho AL\int_0^L \xi_1^2 dx$$
$$\gamma = cL\int_0^L \xi_1^2 dx$$
$$k = \frac{EI}{L^2}\left(\int_0^L \left(\frac{d^2\xi}{dx^2}\right)^2 dx\right) - P\left(\int_0^L \left(\frac{d\xi}{dx}\right)^2 dx\right) + \frac{3EA}{2L^2}\left(\int_0^L \left(\frac{d\xi}{dx}\right)^2 dx\right)^2 (q_{01}+q_{1s})^2$$
$$k_2 = \frac{3EA}{2L^2}\left(\int_0^L \left(\frac{d\xi}{dx}\right)^2 dx\right)^2 (q_{01}+q_{1s}) \quad \text{S20}$$
$$k_3 = \frac{EA}{2L^2}\left(\int_0^L \left(\frac{d\xi}{dx}\right)^2 dx\right)^2$$
$$f = \kappa_d\int_0^L \xi(x)dx$$

Eq. S18 becomes:

$$m\frac{d^2u}{dt^2} + \gamma\frac{du}{dt} + ku + k_2u^2 + k_3u^3 = f \quad \text{S21}$$

By examining carefully the coefficients of Eq. S21, it is noticeable that introducing initial curvature q_{01} to the beam has double impact, as it shifts the value of the linear spring constant (i.e. k) and at the same time creates quadratic nonlinearity (i.e. k_2). This explains why the behavior of arch shaped bi-stable systems is dictated by the quadratic nonlinearity, which always results in softening of the natural frequency[5].

Experimentally, when the actuation power is small, the nonlinear terms in Eq. S21 are negligible and the linear response of the dynamic problem (Figs. 1-4) is mostly dictated by k, as described in the main text. Raising the actuation power and thus increasing the mechanical vibration at a given DC gate voltage, we observe the anticipated softening behavior as well as hysteresis, both before and after the ST transition (Fig. S20). This phenomenon is different from previous results which usually observe hardening at low static loads, and softening at higher gate voltages[6,7].

The solution of Eq. S20 is given by[2,8]:

$$\left[\frac{\gamma^2}{4}+\left(\left(\omega_d-\omega_0\right)-\frac{3}{8}\frac{k_{eff}}{\omega_0}u^2\right)^2\right]u^2=\frac{f_d^2}{4\omega_0^2} \qquad \text{S22}$$

where ω_d and f_d are the frequency and amplitude of the excitation f, respectively, and $k_{eff}=k_3-\frac{9k_2^2}{10\omega_0^2}$ is the effective nonlinear coefficient. We can extract k_{eff} from the resonance shift vs. u^2 at a constant DC voltage, according to the relation[7]:

$$\omega_{\max}-\omega_0=\frac{3}{8}\frac{k_{eff}u^2}{\omega_0} \qquad \text{S23}$$

where $\omega_{\max}$ is the driving frequency for which we detect maximum current, corresponding to maximum deflection. The amplitude u of the vibration is extracted from the maximum current in the frequency response, according to[6]: $I_{peak}=\frac{1}{2}\frac{\partial G}{\partial V_g}\cdot\delta v_{sd}\cdot V_{gDC}\cdot\frac{C_g^{'}}{C_g}\cdot u$.

k_2 linearly depends on the CNT static midpoint displacement $\left(q_{01}+q_{1s}\right)$. Therefore, near the ST point, when the deflection is small, the quadratic nonlinearity is negligible and $k_{eff}\approx k_3$. However, farther from the snap (either before or after), k_2 is expected to be dominant, resulting in softening behavior (k_{eff} < 0) at both low and high static loads. For example, at V_{gDC}=4.8 V (Fig. S11d), we receive an effective nonlinear coefficient of $k_{eff}=\left(\text{-}8.1\pm0.1\right)\cdot10^{32}\ s^{-2}\cdot m^2<0$, in agreement with the observed softening behavior.

Using the k_{eff} and γ coefficients retrieved from the data, we calculated the anticipated response of the system and obtained excellent fit of Eq. S21 to the measured Duffing-like behavior (Figs. S11b and S11d).

This analysis also allows us to estimate the spectral broadening due to symmetry breaking, which has been proposed as an alternative mechanism responsible for the low Q of CNT resonators at room temperature[7]. This geometric symmetry breaking actually refers to the geometric quadratic nonlinearity, k_2. We calculate this effect according to equation S24 of Ref. 7:

$$\Delta f = \frac{3k_{eff}\, k_B T}{8\pi m \omega_0^3} \qquad \text{S24}$$

For example, substituting ω_0 and k_{eff} measured at $V_{gDC} = 4.8V$ into Eq. S24 results in $\Delta f \simeq 0.52\, MHz$ and $Q \simeq 201$, which is 28% smaller than the measured broadening of $\Delta f_{\exp} = 1.86\, MHz$ and yielding Q nearly four times larger than $Q_{\exp} = 56.13$. A similar result is achieved for all five gate voltages marked in Fig. S11a and is plotted in Fig. S12, implying that although characterized by substantial geometric nonlinearity, symmetry breaking is not the major broadening mechanism in our devices.

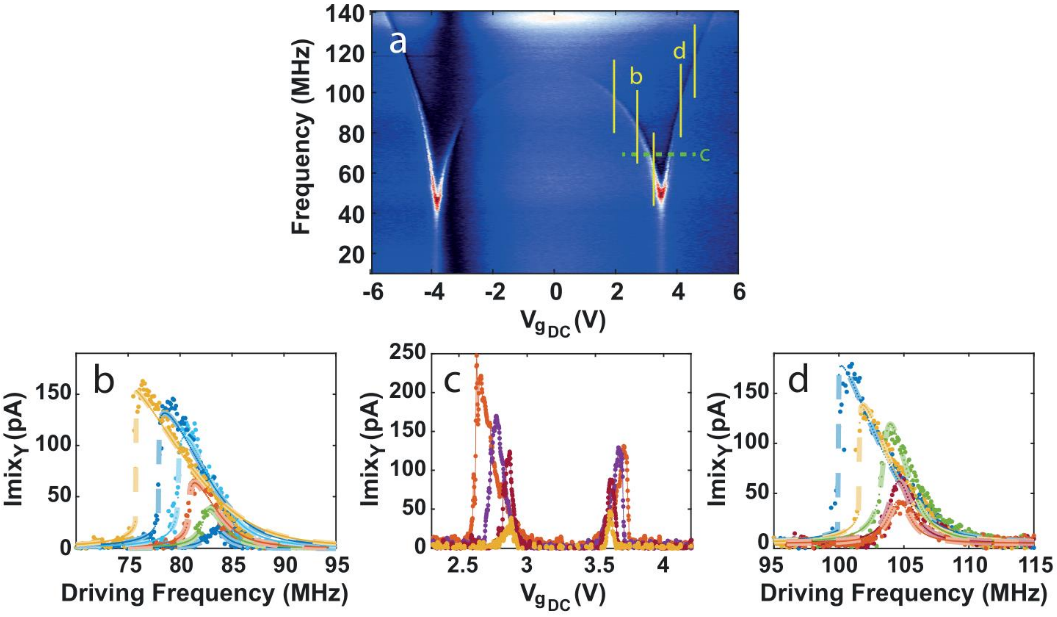

Fig. S11. Duffing-type behavior. (a) Resonance frequency measurement of device I (same as Fig.1c) in the linear regime. (b) and (d) present examples of two out of the five frequency cross sections marked in (a) before (at V_{gDC}=2.5V) and after (at V_{gDC}=4.8V) the IP transition, respectively, for a series of increasing V_{SD} excitations, revealing non-linear dynamic response of the system, governed by softening. Dots are the sampled data and lines are the theoretical fit from solving equation S22. (c) Gate voltage cross sections in low and high excitations (marked by the green line in (a)), also displaying softening behavior.

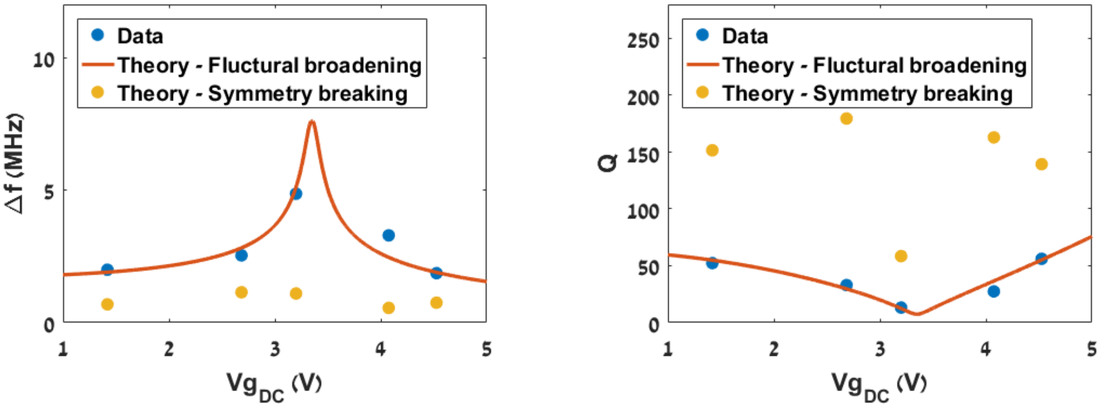

Fig. S12. Comparison between the fluctural broadening and symmetry breaking theories. Peak width (left) and quality factor (right) calculated for device I according to the fluctural broadening theory (Eq. 1, orange line) and according to the symmetry breaking theory (Eq. S24, yellow dots) vs. the experimental data (blue dots).

9. <u>Physical parameters table</u>

Device	Diameter [nm]	Length [μm]	Number of walls
**I**	1.9	0.7	1
**II**	3.5	1.2	2
**III**	1.8	1.34	1
**IV**	2.25	0.86	1
**V**	2	1	1
**VI**	2.6	0.78	1
**VII**	3	1.2	2
**VIII**	1.7	0.9	1
**IX**	2.5	0.89	1
**X**	4	1.3	3

Table S1. Devices physical parameters. Device physical parameters measured in AFM and verified by micro-RAMAN for all devices presented in the text.

Supplementary References

1. Landau, L. D. & Lifshits, E. M. Theory of Elasticity. *Oxford: Pergamon*. Print. (1986).
2. N Nayfeh, A. H. & Mook, D. T. Nonlinear Oscillations. *Weinheim, Germany: Wiley-VCH*. Print. (2004).
3. Barnard, A. W., Sazonova, V., Van Der Zande, A. M. & McEuen, P. L. Fluctuation broadening in carbon nanotube resonators. *Proc. Natl. Acad. Sci. U. S. A.* **109**, 19093–19096 (2012).
4. Zhang, Y. & Dykman, M. I. Spectral effects of dispersive mode coupling in driven mesoscopic systems. *Phys. Rev. B - Condens. Matter Mater. Phys.* **92**, 1–16 (2015).
5. Younis, M. I., Ouakad, H. M., Alsaleem, F. M., Miles, R. & Cui, W. Nonlinear dynamics of MEMS arches under harmonic electrostatic actuation. *J. Microelectromechanical Syst.* **19**, 647–656 (2010).
6. Sazonova, V. *et al.* A tunable carbon nanotube electrochemical oscillator. *Nature* **431**, 284–287 (2004).
7. Eichler, A., Moser, J., Dykman, M. I. & Bachtold, A. Symmetry breaking in a mechanical resonator made from a carbon nanotube. *Nat. Commun.* **4**, 1–7 (2013).
8. Dykman, M. I. & Krivoglaz, M. A. Classical theory of nonlinear oscillators interacting with a medium. *Phys. Status Solidi* **48**, 497–512 (1971).
